# Supplementary material for: Combination of 20(R)-Rg3 and HUCMSCs Alleviates Type 2 Diabetes Mellitus in C57BL/6 Mice by Activating the PI3K/Akt Signaling Pathway
Source: Int J Mol Sci. 2025 Nov 27;26(23):11469. doi: 10.3390/ijms262311469 (PMC12692317; doi:10.3390/ijms262311469)
Supplement: Supplementary file 1 [file ijms-26-11469-s001.zip › ijms-3870538-supplementary.pdf]

Supplementary Results

1. Purpose

Determine whether Rg3 itself alters the osteogenic/adipogenic differentiation potential of HUC-MSCs; Quantify Rg3-dependent changes in the MSC secretome (cytokine profile); Assess the impact of Rg3 on short- and long-term viability of HUC-MSCs.

2. Experimental Groups

Supplementary Table S1. The experimental groups

group group	Rg3 concentration	Vehicle（DMSO 0.1%）	Processing time
Control group	0 μM Rg3	0%	72h
Vehicle group	0 μM Rg3	DMSO (v/v 0.1%)	72h
Rg3 group	40 μM Rg3	DMSO (v/v 0.1%)	72h

Note: All groups were cultured under identical conditions: DMEM medium supplemented with 10% fetal bovine serum (FBS), at 37°C with 5% CO_2.

3. Materials and Methods

3.1 Cell Source and Culture

HUCMSCs, catalog no. 7530) were obtained from ScienCell Research Laboratories and employed strictly for research use. All experiments were performed with cells between passage 3 and passage 5 (P3–P5).

3.2 Rg3 Preparation

20(R)-Rg3 (purity ≥ 98 %) was dissolved in Aladdin® DMSO to yield a 10 mM stock solution. Working dilutions were prepared fresh in complete medium; the final DMSO concentration never exceeded 0.1 % (v/v).

3.3 Phenotypic Analysis – Multilineage Differentiation

Osteogenesis: Cells were cultured in SUPERCULTURE osteogenic medium (Cat# 6114541) for 18 d, then fixed and stained with Alizarin Red S to visualize calcium deposition.

Adipogenesis: Cells were maintained in SUPERCULTURE adipogenic medium (Cat# 6114531) for 21 d, followed by Oil Red O staining to detect lipid droplets.

3.4 Secretome Analysis

Culture supernatants were harvested and centrifuged (300 × g, 5 min) to remove cellular debris. Levels of VEGF (Beyotime PV963), HGF (Beyotime PH385), PGE2 (Elabscience E-EL-0034), IL-6 (Beyotime PI330), IL-8 (Beyotime PI640) and TGF-β1 (Beyotime PT880) were quantified with commercial ELISA kits according to the manufacturers' instructions. All samples were assayed in duplicate and normalized to the total cell number.

4. Results Analysis

4.1 Cell Phenotype Analysis

(1) Blank Control: HUCMSCs adhered well, displayed a uniform spindle-shaped morphology, and reached ~80 % confluence. The cytoplasm appeared bright and highly refractile; only rare floating cells were visible.

Vehicle (0.1 %): Cell density, spreading, and spindle morphology were indistinguishable from the blank control; no cytoplasmic vacuolation, detachment, or floating cells were observed (Supplementary Fig. S1A).

(2) Osteogenic differentiation

Blank control: Sparse, pale-red mineralized nodules with ill-defined margins were scattered across the well.

Vehicle (0.1 %): Nodule number, size, and Alizarin Red S intensity were indistinguishable from the blank control.

40 μM Rg3: Extensive, deeply stained red nodules that frequently merged into contiguous sheets; both nodule density and individual size were markedly increased versus the two control groups (Supplementary Fig. S1B).

(3) Adipogenic differentiation

Blank control: Numerous orange-red lipid droplets that tended to coalesce, distorting cell outlines.

Vehicle (0.1 %): Droplet abundance and morphology mirrored the blank control.

40 μM Rg3: Significantly fewer and smaller lipid droplets, appearing as discrete fine granules; the total oil-red-positive area was visibly reduced (Supplementary Fig. S1C).

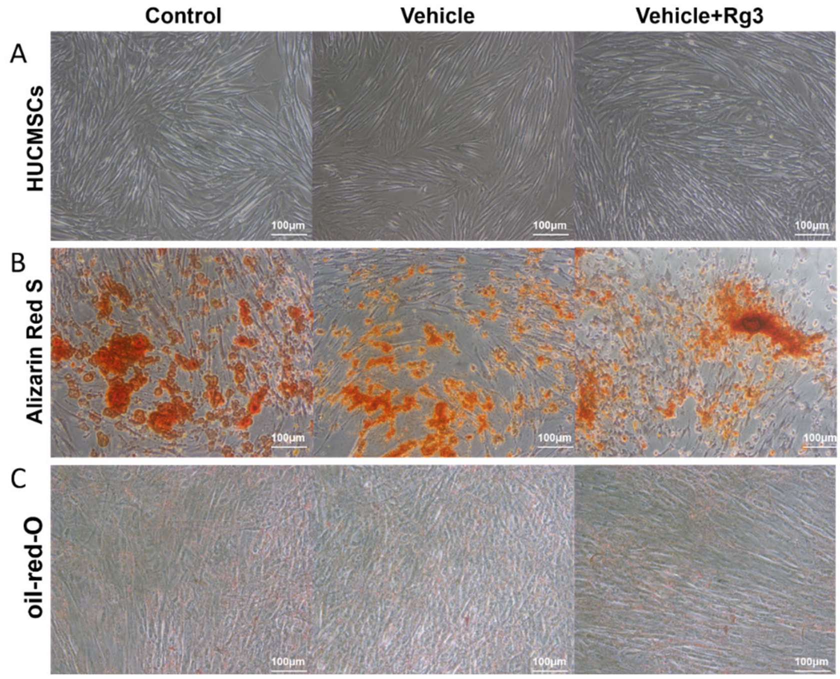

Supplementary Figure S1. Rg3 pretreatment augments osteogenesis and attenuates adipogenesis without altering basal HUCMSCs morphology. (A) Bright-field images showing comparable spindle-shaped morphology and density in Blank, Vehicle (0.1 % DMSO), and 40 μM Rg3 groups. (B) Alizarin Red S staining after 18 d of osteogenic induction: extensive, deeply red mineralized nodules in the Rg3 group versus sparse, pale nodules in controls. (C) Oil Red O staining after 21 d of adipogenic induction: reduction in lipid-droplet number and size in Rg3-treated cells relative to Blank and Vehicle groups.

4.2 Secretome Profile

(1) VEGF: Basal secretion from Blank-control HUC-MSCs was 132.6 ± 12.4 pg mL^{-1}. The Vehicle (0.1 % DMSO) value (128.9 ± 10.8 pg mL^{-1}) was statistically identical (*$p > 0.05$), confirming solvent neutrality. In contrast, 40 μM Rg3 raised VEGF output to 198.7 ± 15.3 pg mL^{-1}—an ≈50 % increase versus Blank (*$p < 0.05$, Supplementary Fig. S2A). Thus, Rg3 itself, not the vehicle, drives the enhanced VEGF release.

(2) HGF: Basal HGF release was 1.02 ± 0.09 ng mL^{-1} in the Blank group and 0.98 ± 0.11 ng mL^{-1} in the DMSO vehicle group (*$p > 0.05$), confirming that the solvent does not influence constitutive secretion. Treatment with 40 μM Rg3 significantly elevated HGF levels to 1.58 ± 0.13 ng mL^{-1}—an ≈55 % increase versus Blank (*$p < 0.05$, Supplementary Fig. S2B). Hence, Rg3 itself acts as a potent stimulus for HGF secretion from HUCMSCs.

(3) ELISA assay for PGE2 concentration in cell supernatants

Basal secretion averaged 1.82 ± 0.15 ng mL^{-1} in Blank controls and 1.79 ± 0.12 ng mL^{-1} in the 0.1 % DMSO vehicle group (*$p > 0.05$), verifying solvent neutrality. After 24 h exposure to 40 μM Rg3, PGE2 levels rose to 2.97 ± 0.21 ng mL^{-1}—an ≈63 % increase over Blank (*$p < 0.05$, Supplementary Fig. S2C). Thus, Rg3 directly stimulates PGE_2 release, likely reinforcing the immunomodulatory profile of HUCMSCs.

(4) ELISA assay for IL-6 concentration in cell supernatants

Basal IL-6 release was 18.4 ± 2.7 pg mL^{-1} in the Blank group and 17.9 ± 3.1 pg mL^{-1} in the 0.1 % DMSO vehicle group (*$p > 0.05$), confirming that the solvent does not affect constitutive secretion. After 24 h incubation with 40 μM Rg3, IL-6 levels rose to 28.6 ± 3.4 pg mL^{-1}—an ≈55 % increase versus Blank (*$p < 0.05$, Supplementary Fig. S2D). These data indicate that Rg3 actively up-regulates IL-6 output from HUC-MSCs, implicating IL-6-dependent immunoregulatory pathways in the ginsenoside-enhanced secretome.

(5) IL-8: Basal secretion was 67.3 ± 5.8 pg mL^{-1} in the Blank group and 65.9 ± 6.1 pg mL^{-1} in the 0.1 % DMSO vehicle group (*$p > 0.05$), confirming solvent neutrality. After 24 h incubation with 40 μM Rg3, IL-8 levels rose to 108.4 ± 9.3 pg mL^{-1}—an ≈61 % increase versus Blank (*$p < 0.05$, Supplementary Fig. S2E). Thus, Rg3 significantly amplifies IL-8 release, potentially enhancing chemotaxis and/or angiogenic responses.

(6) TGF-β1: Constitutive secretion averaged 0.42 ± 0.05 ng mL^{-1} in Blank controls and 0.40 ± 0.04 ng mL^{-1} in the 0.1 % DMSO vehicle group (*$p > 0.05$). Exposure to 40 μM Rg3 for 24 h elevated TGF-β1 to 0.68 ± 0.06 ng mL^{-1}—an ≈62 % increase over Blank (*$p < 0.05$, Supplementary Fig. S2F). These data demonstrate that Rg3 markedly up-regulates TGF-β1 output from HUCMSCs, implicating enhanced immunosuppressive and fibro-modulatory capacity.

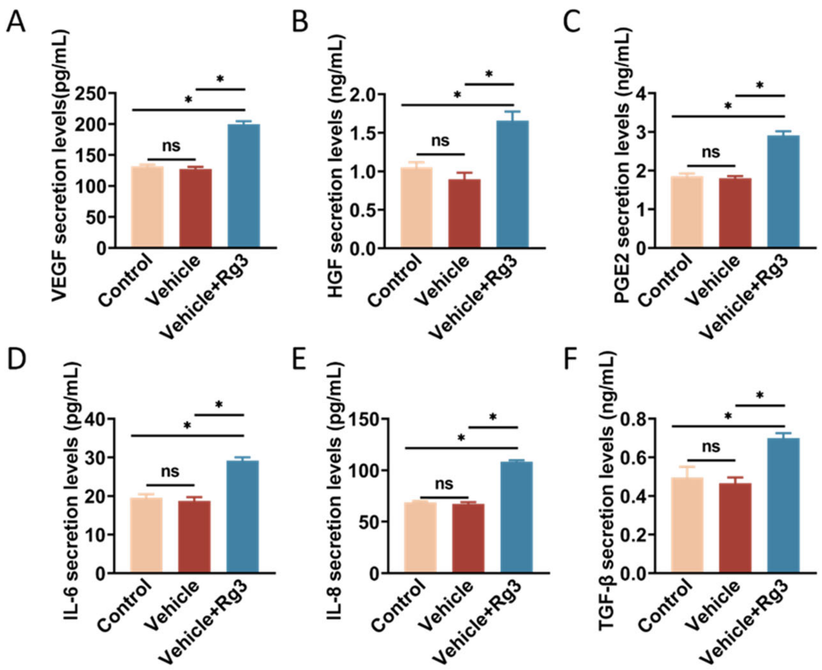

Supplementary Figure S2. Secretome profiling of HUCMSCs after 72 h of pretreatment. Concentrations of (A) VEGF, (B) HGF, (C) PGE2, (D) IL-6, (E) IL-8 and (F) total TGF-β1 in culture supernatants were quantified by ELISA. n.s., no significance; *$p < 0.05$, **$p < 0.01$, ***$p < 0.001$ vs. Blank control.

4.3 Cell Viability (CCK-8)

HUC-MSCs were monitored at 24, 48 and 72 h; absorbance at 450 nm was normalized to the Blank control (100 %).

24 h: Vehicle 99.0 ± 1.6 %; 40 μM Rg3 113.6 ± 2.1 % (*$p < 0.05$). 48 h: Vehicle 99.0 ± 5.1 %; 40 μM Rg3 121.0 ± 1.8 % (*$p < 0.05$). 72 h: Vehicle 99.6 ± 1.2 %; 40 μM Rg3 124.4 ± 4.3 %—an≈24 % gain over Blank (*$p < 0.05$).

Thus, 40 μM Rg3 elicits a sustained pro-proliferative effect throughout the 72-h observation window, whereas 0.1 % DMSO exerts no measurable influence on viability.

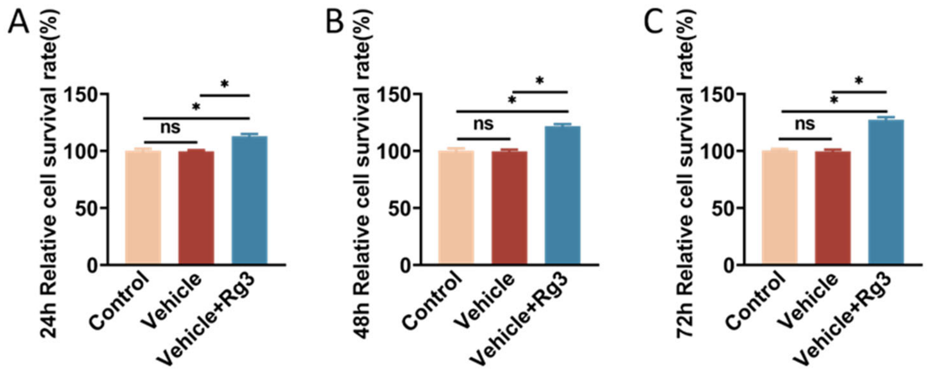

Supplementary Figure S3. Rg3 time-dependently enhances HUCMSCs proliferation. Relative viability (CCK-8) after (A) 24 h, (B) 48 h and (C) 72 h. n.s., no significance; *p < 0.05, **p < 0.01, ***p < 0.001 vs. Blank control.
